# The Patients' Column

**Published:** 1914-04-18

**Authors:** 


### The Patients' Column.

IN A PROVINCIAL ISOLATION HOSPITAL.

[The following is a brief account of an experience, which we publish here, under the above heading, as being one of a series of less critical " patients' points of view." The interest of these is rather in the attitude of mind revealed than in the exercise of a keen critical faculty.]

" In briefly giving my experiences of institutional treatment, I must at the outset state that I had never previously entered a hospital of any description, so when I consented to be taken to the isolation hospital, suffering from diphtheria, I did so with much reluctance and many misgivings. I was agreeably surprised, on arrival, to find myself in an up-to-date ward of decidedly comfortable appearance, treated in a pretty blue, with polished doors of pitch pine, and furnishing which betokened every comfort. I was at once put to bed, and antitoxin injected by the matron. For the first few days I was kept on milk and beef-tea, and a close watch taken of my pulse and temperature, which was carefully charted night and morning. It was pleasing to note that the thermometer was always washed before being passed from one patient to another. Flowers and plants were removed from the ward at night, and one could not fail to notice how spotlessly clean everything was, which, however, could readily be understood, for the matron's keen eye was always on the look-out for any remissness on the part of the wardmaid. There were four beds in my ward, my companions being children. The meals were admirably cooked and dished up, and served in a manner calculated to tempt the most delicate appetite, and a patient could always have more for the asking. The hospital was a new and up-to-date one, consisting of four wards (four beds in each), two ward kitchens, verandahs, and usual offices, and possessed its own gas-house (acetylene), filter-beds, and laundry, whilst some considerable distance away, under the same administration, were the sanatorium and smallpox hospital. Strange to relate, however, no bathroom had been provided, and one had to take a bath in the ward whilst the children were asleep. Of course, a small screen was provided, but when convalescent one would much prefer the privacy of a bathroom. The bath itself was an enamelled one on wheels, and when filled was as much as two nurses could do to drag it into the ward. If this should catch the eye of the authorities concerned, it is to be hoped this omission will soon be rectified. The provision against fire—a bucket of water in each ward—appeared quite inadequate. I left the hospital more than satisfied with the treatment received, and fully convinced as to the necessity for such institutions."

